# Recommendations for the Screening of Breast Cancer of the Brazilian College of Radiology and Diagnostic Imaging, Brazilian Society of Mastology and Brazilian Federation of Gynecology and Obstetrics Association

**DOI:** 10.1055/s-0043-1772498

**Published:** 2023-09-08

**Authors:** Linei Augusta Brolini Delle Urban, Luciano Fernandes Chala, Ivie Braga de Paula, Selma di Pace Bauab, Marcela Brisighelli Schaefer, Ana Lúcia Kefalás Oliveira, Carlos Shimizu, Tatiane Mendes Gonçalves de Oliveira, Paula de Camargo Moraes, Beatriz Medicis Maranhão Miranda, Flávia Engel Aduan, Salete de Jesus Fonseca Rego, Ellyete de Oliveira Canella, Henrique Lima Couto, Gustavo Machado Badan, José Luis Esteves Francisco, Thaís Paiva Moraes, Rosangela Requi Jakubiak, João Emílio Peixoto

**Affiliations:** 1 Brazilian College of Radiology and Diagnostic Imaging, São Paulo, SP, Brazil; 2 National Mammography Commission, Brazilian College of Radiology and Diagnostic Imaging, São Paulo, SP, Brazil; 3 National Mammography Commission, Representative of the Brazilian Society of Mastology, São Paulo, SP, Brazil; 4 National Mammography Commission, Representative of the Brazilian Federation of Associations of Gynecology and Obstetrics, São Paulo, SP, Brazil

**Keywords:** breast cancer screening, mammography, ultrasound, magnetic resonance imaging, rastreamento de câncer de mama, mamografia, ultrassom, imagem de ressonância magnética

## Abstract

**Objective** To present the update of the recommendations of the Brazilian College of Radiology and Diagnostic Imaging, the Brazilian Society of Mastology and the Brazilian Federation of Associations of Gynecology and Obstetrics for breast cancer screening in Brazil.

**Methods** Scientific evidence published in Medline, EMBASE, Cochrane Library, EBSCO, CINAHL and Lilacs databases between January 2012 and July 2022 was searched. Recommendations were based on this evidence by consensus of the expert committee of the three entities.

**Recommendations** Annual mammography screening is recommended for women at usual risk aged 40–74 years. Above 75 years, it should be reserved for those with a life expectancy greater than seven years. Women at higher than usual risk, including those with dense breasts, with a personal history of atypical lobular hyperplasia, classic lobular carcinoma in situ, atypical ductal hyperplasia, treatment for breast cancer or chest irradiation before age 30, or even, carriers of a genetic mutation or with a strong family history, benefit from complementary screening, and should be considered individually. Tomosynthesis is a form of mammography and should be considered in screening whenever accessible and available.

## Resumo

**Objetivo** Apresentar a atualização das recomendações do Colégio Brasileiro de Radiologia e Diagnóstico por Imagem da Sociedade Brasileira de Mastologia e da Federação Brasileira das Associações de Ginecologia e Obstetrícia para o rastreamento do câncer de mama no Brasil.

**Métodos** Foram pesquisadas evidências científicas publicadas nas bases de dados Medline EMBASE Biblioteca Cochrane EBSCO CINAHL e Lilacs entre janeiro de 2012 e julho de 2022. As recomendações foram baseadas nessas evidências por consenso do comitê de especialistas das três entidades.

**Recomendações** A mamografia anual é recomendada para mulheres com risco habitual entre 40 e 74 anos. Acima de 75 anos deve ser reservado para aqueles com expectativa de vida superior a sete anos. Mulheres com risco maior do que o normal incluindo aquelas com mamas densas com história pessoal de hiperplasia lobular atípica carcinoma lobular in situ clássico hiperplasia ductal atípica tratamento para câncer de mama ou irradiação de tórax antes dos 30 anos ou ainda portadoras de doença genética mutação ou com forte histórico familiar beneficiam-se de triagem complementar e devem ser considerados individualmente. A tomossíntese é uma forma de mamografia e deve ser considerada na triagem sempre que acessível e disponível.

## Introduction

In 2021, breast cancer became the most frequently diagnosed cancer in the world, and the main cause of premature death in women. 1 In Brazil, 73,610 new cases of breast cancer were estimated for the year 2023, which represents an adjusted incidence rate of 41.89 cases per 100,000 women. 1 Screening is an effective measure to detect the disease at an early stage and reduce its mortality. In addition, the early diagnosis of breast cancer allows for a greater range of therapeutic options and a reduction in treatment morbidity. 2 3 4

In 2012 and 2017, the Brazilian College of Radiology and Diagnostic Imaging (CBR), the Brazilian Society of Mastology (SBM) and the Brazilian Federation of Associations of Gynecology and Obstetrics (Febrasgo), through the National Mammography Commission (CNM), published recommendations for breast cancer screening. 5 6 The purpose of this update is to publish the available evidence on screening and provide information for decision-making in women at different risks for developing the disease.

## Methods

Searches were performed in the Medline (via PubMed), EMBASE, Cochrane Library, EBSCO, CINAHL and Lilacs (via Bireme) databases using as many keywords, descriptors and MeSH terms as possible to find scientific evidence of breast cancer screening with mammography, ultrasound (US), magnetic resonance imaging (MRI) and tomosynthesis (TS) in women at usual, intermediate and high risk for breast cancer, published between January 2012 and July 2022 in Portuguese, English, French and Spanish. Complementary searches were performed on Web sites, online tools and in the references of the analyzed studies. The most recent, higher quality evidence processed (systematic reviews and meta-analyses) that better answered the structured questions were selected for analysis. In the absence of these, primary studies (clinical trials or cohorts) were included. The risk of bias in the studies was assessed using the following tools: ROBIS (Risk of Bias in Systematic Reviews), RoB 2.0 (Cochrane Risk of Bias Tools for Randomized Controlled Trials version 2.0), QUADAS-C (Quality Assessment of Diagnostic Accuracy Studies – Comparative) and ROBINS-I (Risk of Bias in Non-randomized Studies of Interventions). The overall quality of the evidence set for each outcome was assessed using GRADE (Grading of Recommendations Assessment, Development and Evaluation).

The recommendations were based on this evidence through consensus of the committee of experts from the three entities (CBR, SBM and Febrasgo), defined when the members reached at least 75% agreement with the recommendation. In the absence of an initial agreement, in a second round of discussion and voting, a simple majority was needed to define consensus. The recommendations were classified into five categories:

- **Category A** – **Strong** recommendation **in favor** based on **high-quality** evidence.
- **Category B** – **Strong** recommendation **in favor** based on **moderate-quality** evidence.
- **Category C** – **Weak** recommendation **in favor** based on **low-quality** evidence.
- **Category D** – Recommendation **in favor** , based only on **expert** consensus.
- **Category E** – Recommendation **against** as there is insufficient evidence to support its use.

## Screening Recommendations

### Screening of Women at Usual Population Risk

- ***Mammography:***
  ○ Annual mammography screening is recommended for women aged 40–74 years, preferably with digital technology (Category A).
  ○ From the age of 75, it is recommended to continue screening if there are no comorbidities that reduce life expectancy and if any, life expectancy should be of at least seven years (Category D).
- ***Ultrasound:***
  ○ US is not recommended as supplementary screening or as an isolated method for women at usual risk (Category E).
○ *Note: the use of US is considered in specific higher risk situations (see section on dense breasts, intermediate risk and high risk).*
- ***Magnetic resonance imaging:***
  ○ MRI is not recommended as supplemental screening or as an isolated method for women at usual risk (Category E).
○ *Note: the use of MRI is considered in specific higher risk situations (see section on dense breasts, intermediate risk and high risk).*
- ***Tomosynthesis:***
  ○ It is recommended to consider TS in combination with synthesized mammography (SM) or standard mammography (combination mode) in screening when affordable and available (Category B).
  ○ It is recommended to consider TS in combination with synthesized 2D mammography (SM) or standard 2D mammography (combination mode) in screening when affordable and available (Category B).

### Screening of Women with Dense Breasts

- ***Mammography:***
  ○ Annual screening with mammography is recommended for women aged 40–74 years, preferably with digital technology (Category A).
  ○ From the age of 75, it is recommended to continue screening if there are no comorbidities that reduce life expectancy and, if any, life expectancy should be of at least seven years (Category D).
- ***Ultrasound:***
  ○ It is recommended to consider annual US as an adjunct to mammography in women with dense breasts, except when MRI is performed (Category B).
- ***Magnetic resonance imaging:***
  ○ It is recommended to consider biennial MRI as an adjunct to mammography in extremely dense breasts (Category C).
- ***Tomosynthesis:***
  ○ It is recommended to consider TS in combination with synthesized 2D mammography (SM) or standard 2D mammography (combination mode) in screening when affordable and available (Category B).

### Screening of Women with a Personal Biopsy History of Atypical Lobular Hyperplasia (ALH), Classic Lobular Carcinoma in Situ (LCIS), and Atypical Ductal Hyperplasia (ADH)

- ***Initial remark:***
  ○ It is recommended to evaluate women with ALH, LCIS or ADH by risk calculation models that include these variables in conjunction with other clinical data, including family history and breast density, to estimate breast cancer risk.
- ***Mammography:***
  ○ For women with estimated lifetime risk < 20%, annual mammography is recommended from age 40 (Category A).
  ○ For women with estimated lifetime risk ≥ 20%, annual mammography is recommended from diagnosis (not before age 30) (Category B).
- ***Ultrasound:***
  ○ For women with an estimated 15–20% lifetime risk, US can be considered as an adjunct to mammography (Category D).
  ○ For women with an estimated lifetime risk ≥ 20%, US is recommended as an alternative method for those who, for whatever reason, cannot undergo MRI (Category B).
- ***Magnetic resonance imaging:***
  ○ For women with estimated lifetime risk ≥ 20%, annual MRI should be considered as an adjunct to mammography from diagnosis (not before age 25) (Category B).
- ***Tomosynthesis:***
  ○ It is recommended to consider TS in combination with synthesized 2D mammography (SM) or standard 2D mammography (combination mode) in screening when affordable and available (Category B).

### Screening of Women with a Personal History of Treatment for Invasive Breast Cancer or Ductal Carcinoma in Situ (DCIS)

- ***Mammography:***
○ Women treated with **conservative surgery** should undergo mammography annually (Category A), starting at least six months after the end of radiotherapy.
○ Women treated with **mastectomy** should undergo annual mammography of the contralateral breast only, starting one year after the end of treatment (Category A).
○ Women undergoing **adenomastectomy** may consider performing mammography within one year to assess residual fibroglandular tissue to determine the need for continued mammographic screening (Category D).
- ***Ultrasound:***
  ○ US can be used in complementary screening to mammography when MRI is indicated but for whatever reason cannot be performed (Category C).
- ***Magnetic resonance imaging:***
○ Women treated with **conservative surgery** or **mastectomy** (to evaluate the contralateral breast) who were diagnosed with breast cancer before age 50 or with dense breasts should undergo annual MRI (Category C), starting one year after the end of treatment.
- ***Tomosynthesis:***
  ○ It is recommended to consider TS in combination with synthesized 2D mammography (SM) or standard 2D mammography (combination mode) in screening when affordable and available (Category B).

### Screening of Women with a Personal History of Chest Radiotherapy

- ***Mammography:***
○ Women with a history of chest irradiation before the age of 30 should undergo mammography **annually from the eighth year after radiotherapy treatment (not before age 30)** (Category A).
- ***Ultrasound:***
  ○ US should be used for screening only when MRI, for whatever reason, cannot be performed (Category B).
- ***Magnetic resonance imaging:***
○ Women with a history of chest irradiation before the age of 30 should undergo **MRI annually from the eighth year after radiotherapy treatment (not before age 25)** (Category A).
- ***Tomosynthesis:***
  ○ It is recommended to consider TS in combination with synthesized 2D mammography (SM) or standard 2D mammography (combination mode) in screening when affordable and available (Category B).

### Screening of Women with a Genetic Mutation or a Strong Family History of Breast Cancer (Lifetime Risk ≥ 20%)

- ***Mammography:***
○ Women with a pathogenic mutation of the BRCA1 gene or not tested, but with first-degree relatives who are carriers should undergo mammography **annually from the diagnosis of the mutation (not before age 35)** (Category A).
○ Women with a pathogenic mutation of the TP53 gene or not tested, but with first-degree relatives who are carriers should undergo mammography **annually from the diagnosis of the mutation (not before age 30)** (Category A).
○ Women with a pathogenic mutation of the BRCA2 gene or other genes at moderate or high risk for breast cancer, in addition to those not tested but with first-degree relatives who are carriers should undergo mammography **annually after the diagnosis of the mutation (not before age of 30)** (Category A).
○ Women with a lifetime risk ≥ 20%, as calculated by one of the mathematical models based on family history should undergo mammography **annually, starting 10 years before the youngest relative's age of diagnosis (not before age 30)** (Category A).
- ***Ultrasound:***
  ○ US should be used for screening only when MRI, for whatever reason, cannot be performed (Category B).
- ***Magnetic resonance imaging:***
  ○ Women with a pathogenic mutation of the BRCA1 gene or not tested, but with first-degree relatives who are carriers should undergo MRI annually from the diagnosis of the mutation (not before age 25) (Category A).
  ○ Women with a pathogenic mutation of the TP53 gene or not tested, but with first-degree relatives who are carriers should undergo MRI annually from the diagnosis of the mutation (not before age 20) (Category A).
  ○ Women with a pathogenic mutation of the BRCA2 gene or other genes at moderate or high risk for breast cancer, in addition to those not tested, but with first-degree relatives who are carriers should undergo MRI annually from the diagnosis of the mutation (not before age 30) (Category A).
  ○ Women with a lifetime risk ≥ 20% calculated by one of the mathematical models based on family history should undergo MRI annually, starting 10 years before the youngest relative's age of diagnosis (not before age 30).
- ***Tomosynthesis:***
  ○ It is recommended to consider TS in combination with synthesized 2D mammography (SM) or standard 2D mammography (combination mode) in screening when affordable and available (Category B).

## Rationale

The benefits of mammographic screening have been evaluated using cohort studies, systematic reviews and randomized clinical trials, demonstrating a reduction of 22–30% in specific mortality from breast cancer in women aged 40 to 74 years. 2 3 4 7 When other important outcomes were analyzed, a better quality of life measured using the QALY (quality-adjusted life-years) was also observed, given the less aggressive treatments, 2 in addition to a higher rate of initial tumors with better prognostic characteristics and negative axilla, 3 and 28% fewer advanced tumors. 4

## Starting Age and Frequency of Screening

Starting screening at age 40 reduces 10-year mortality from breast cancer by 25%, but increases false-positive rates from 4.8% to 7%. 7 In Brazil, 41.1% of women diagnosed with breast cancer are younger than 50 years. 8 Regarding the screening interval, the two-year interval is related to a higher risk of advanced tumors (RR: 1.28), larger than 15 mm and with worse prognostic factors. 7 Thus, the CNM recommends annual mammography screening starting at age 40.

## Considerations for Women under 40

Screening in this age group is not recommended given the lower incidence of breast cancer (∼7% of cases). However, the AMAZONA III study showed this number is 17% in Brazil, with larger tumors and worse prognosis at diagnosis compared with women over 40 years of age. 9 Therefore, in agreement with other international societies, 10 11 the CNM recommends that the attending physician performs an assessment of the estimated risk of breast cancer for all women over 30 years of age using mathematical models to better stratify those at high risk, who could benefit from differentiated screening.

## When to Stop Screening

As prospective, controlled and randomized studies did not include women over 74 years of age, direct data on screening in this age group are not available. However, the life expectancy of women has increased, with an increasing incidence of breast cancer in the age group above 75 years. Currently, 26% of deaths from breast cancer occur in women diagnosed after the age of 74. 12 13 Considering these factors, many medical organizations recommend individualizing the decision that should be discussed with the woman.

## Adverse Effects of Screening

Although some adverse effects are reported, the quality of evidence for analyzing them is low. Overdiagnosis is a debated effect, but its estimation is variable given the difficulty in determining which tumor would or would not cause the patient's death. 14 The risk of carcinoma induced by the radiation used in mammographic screening is low, although higher in women with large breasts, in whom the radiation dose is higher, as well as in those undergoing supplemental incidences. 15 It was also associated with a 2.9% increase in the risk of biopsies with benign lesions, which can cause anxiety. 14 However, the reduction in mortality of cancer detected early by screening outweighs the risks of damage caused by exposure to radiation.

## Considerations about Breast Tomosynthesis

TS is an evolution of the digital mammography. Numerous studies confirm the effectiveness of this technology in breast cancer screening, which increases the detection rate by up to 50%, 16 17 18 19 20 and reduces the recall rate for additional images by 9% to 29%. 19 20 The detected tumors have histological and immunohistochemical characteristics similar to those detected by mammography, 21 22 23 and results are maintained in subsequent rounds. 24 Therefore, TS is recommended by the CNM as a screening method when accessible and available, as well as by various medical societies, including the *American College of Radiology* (ACR), 10 the *American Cancer Society* (ACS), 25 the *European Society of Breast Imaging* (EUSOBI), 26 the *Société d'Imagerie de la Femme* (SIFEM), 27 the *National Comprehensive Cancer Network* (NCCN) 11 and the *European guidelines on breast cancer screening and diagnosis* . 28

Tomosynthesis should be used in combination with standard 2D mammography (combination mode) or with synthesized 2D mammography (SM); the latter has the advantage of reducing the radiation dose. 15 17 18 As the National Health Surveillance Agency (Anvisa) has not established the reference and tolerance levels of the glandular dose for TS in Brazil yet, the recommendation is that each service should carry out a survey of the mean glandular doses using a sample of patients with breasts of different thickness, thereby establishing local reference and tolerance levels. 29 30

## Screening Considerations for Women with Dense Breasts

Dense breast is a risk factor for breast cancer and associated with reduced mammographic sensitivity. For these reasons, supplementary methods have been proposed. All supplemental modalities have improved sensitivity over mammography alone, allowing the detection of early-stage cancers hidden in mammograms. 31 32 33 34 35 36 37 38

Magnetic resonance imaging is the supplementary technique with the highest rate of additional cancer detection. 31 This increases the likelihood of less invasive and curative treatments. Data on critical outcomes such as mortality are not available. However, randomized trials have shown that the supplemental use of US in dense breasts and MRI in extremely dense breasts reduced the rate of interval cancer, an important patient-centered surrogate outcome. 24 34 39 Regarding harm, the use of supplemental modalities is associated with increased false positives and biopsies. 31 33 35 36 37 38 Thus, for women with dense breasts without other risk factors, the CNM recommends annual mammography screening starting at age 40, with the option of using supplementary methods such as US or MRI. For extremely dense breasts, there is scientific evidence suggesting the superiority of MRI.

## Screening Considerations for Women with a Personal History of ALH, LCIS, and ADH Diagnosis

Atypical ductal hyperplasia, ALH and LCIS are considered non-obligate precursor lesions for DCIS and invasive carcinomas, 40 and confer an increased relative risk for their subsequent development throughout life, ranging from 2.6–5.0 times for ADH, 3.2–4.8 times for ALH and 6–10 times for LICS. 41 42 43 44 45 46 47 48 49

Studies evaluating screening in this group are scarce and based on retrospective series that estimated the risk for in situ and subsequent invasive carcinomas. The current strategy for defining screening in this subgroup is based on calculating the lifetime risk for breast cancer. 11 Factors such as age at diagnosis and breast density directly impact the risk of cancer, which can be estimated using risk calculation tools based on mathematical models. 47 Currently, few models include this group in the risk calculation, namely the Breast Cancer Risk Assessment Tool and the IBIS Breast Cancer Risk Evaluation Tool, and these should be preferably used. 11 47

## Screening Considerations for Women with a Personal History of Treatment for Invasive Breast Cancer and DCIS

Women with a personal history of breast cancer are seven times likelier to develop a second malignant neoplasm in the ipsilateral or contralateral breast. 48 In patients treated with conservative surgery, mammography is less sensitive because of the surgical alterations and higher incidence of interval carcinoma, 49 which explains the need for additional screening.

Complementary screening with MRI can detect 8.2–18.1 additional cancers to mammography per 1,000 women. 50 51 52 53 54 55 The performance of MRI in this scenario has shown to be similar to that of patients at high genetic risk, considering the sensitivity, detection rate, false positive and positive predictive value (PPV) of biopsies. 56 57 58 However, the scientific evidence for MRI in this population is weak, based on predominantly retrospective studies. 49 50 55 56 57 58 59 Among this heterogeneous group, the benefit of MRI is better established in young patients (diagnostic age < 50 years) and with dense breasts. 49 50 51 52

Few studies have evaluated the accuracy of US, with a detection rate of additional cancers to mammography of 2.4 to 4.3/1,000 women, but with an increase in false positives and lower PPV for biopsies. When performed in addition to MRI, US does not improve sensitivity, 53 54 but it can be used as supplemental screening when MRI is not available.

In patients with a personal history of breast cancer treated with mastectomy, imaging screening of the treated breast with or without reconstruction is not indicated given the low detection rate of asymptomatic cancers by mammography, US or MRI. 59

## Screening Considerations for Women with a History of Thoracic Radiotherapy

Women treated with thoracic radiotherapy before the age 30 have a 13.4 times higher average risk of developing breast cancer than the general population, similar to those carrying the BRCA1 gene mutation. 60 The increased incidence occurs ∼10 years after treatment, persisting 30 years later. The highest incidence occurs when treatment is performed at 10–14 years of age (RR = 22.0) and 15–19 years of age (RR = 14.3). 61 For this group, there is evidence of the importance of screening with mammography and MRI starting at 25 years of age or eight years after radiotherapy, in accordance with the recommendations of other medical entities, such as the Children's Oncology Group and the International Guideline Group. 60

## Screening of Women with a Genetic Mutation or a Strong Family History of Breast Cancer (Lifetime Risk ≥ 20%)

Mutations in genes that predispose to breast cancer are classified as high risk, when they cause an increase of five times or more in relation to non-carrier women (BRCA1, BRCA2, TP53, PTEN, among others), or intermediate risk, when they increase 1.5–5 times (ATM, CHECK2, BARD1, among others). 62 63 64 In Brazil, a study demonstrated that the most common mutation genes were BRCA1 (27.4%), BRCA2 (20.3%), TP53 (10.5%), ATM (8.8%), CHEK2 (6.2%) and PALB2 (5.1%). 64 The Brazilian variant TP53 R337H was strongly associated with the risk of breast cancer (OR = 17.4). 64 In the case of women with a strong family history of breast cancer but without known mutation, those with an estimated **≥ 20% lifetime risk** calculated by mathematical models were defined as high risk. 62 These women have the cancer at an early age, with peak incidence at 20–35 years for the PT53 mutation, 30–39 years for the BRCA1 mutation, 30–49 years for BRCA2 mutations, and 40–59 years for the high familial risk. 62 63 64 65

For this risk group, there is strong scientific evidence of the importance of MRI screening because of the reduction of interval cancers and the higher detection rate of tumors in early stages, which may reduce the need for chemotherapy and mortality, despite the higher number of false positives. 54 55 65 66 67 As for mammography, its role in patients with BRCA1 mutation has recently been questioned. A meta-analysis 68 demonstrated that the addition of mammography to MRI in patients with BRCA1 mutation modestly increased sensitivity (3.99%) and reduced specificity (4%). As for the BRCA2 mutation, the increase in sensitivity was greater (12.6%), with a small reduction in specificity (5%). Thus, the CNM recommends screening with MRI, associated with mammography, but not starting mammography before age 35 for BRCA1 and 30 for the other groups. Additional US examinations do not yield additional detection of cancer if MRI is performed and should be reserved for further evaluation or to guide biopsy of findings identified on MRI.

As for the impact on mortality, an important study was published by Bae et al. 54 Even though this was a retrospective study, it was demonstrated that high-risk women screened with mammography and MRI had better overall survival and tumors diagnosed at stages of better prognosis than patients in the mammography-only group.

## Conclusion

This guideline brought the consensus of recommendations based on current data for breast cancer screening in Brazil, subdivided into sections according to the risk for developing breast cancer, from women at usual risk, who represent ∼80% of patients diagnosed with breast cancer, to women at higher risk.
